# A highly conserved cryptic epitope in the receptor binding domains of SARS-CoV-2 and SARS-CoV

**DOI:** 10.1126/science.abb7269

**Published:** 2020-04-03

**Authors:** Meng Yuan, Nicholas C. Wu, Xueyong Zhu, Chang-Chun D. Lee, Ray T. Y. So, Huibin Lv, Chris K. P. Mok, Ian A. Wilson

**Affiliations:** 1 Department of Integrative Structural and Computational Biology, The Scripps Research Institute, La Jolla, CA 92037, USA.; 2 HKU-Pasteur Research Pole, School of Public Health, Li Ka Shing Faculty of Medicine, The University of Hong Kong, Hong Kong SAR, China.; 3 The Skaggs Institute for Chemical Biology, The Scripps Research Institute, La Jolla, CA, 92037, USA.

## Abstract

The outbreak of coronavirus disease 2019 (COVID-19) caused by severe acute respiratory syndrome–coronavirus 2 (SARS-CoV-2) has now become a pandemic, but there is currently very little understanding of the antigenicity of the virus. We therefore determined the crystal structure of CR3022, a neutralizing antibody previously isolated from a convalescent SARS patient, in complex with the receptor binding domain (RBD) of the SARS-CoV-2 spike (S) protein at 3.1-angstrom resolution. CR3022 targets a highly conserved epitope, distal from the receptor binding site, that enables cross-reactive binding between SARS-CoV-2 and SARS-CoV. Structural modeling further demonstrates that the binding epitope can only be accessed by CR3022 when at least two RBDs on the trimeric S protein are in the “up” conformation and slightly rotated. These results provide molecular insights into antibody recognition of SARS-CoV-2.

## Targeting the SARS-CoV-2 spike

The surface of severe acute respiratory syndrome–coronavirus 2 (SARS-CoV-2) is decorated with trimeric spikes that bind to host cell receptors. These spikes also elicit an antibody response, so understanding antibody recognition may aid in vaccine design. Yuan *et al.* determined the structure of CR3022, a neutralizing antibody obtained from a convalescent SARS-CoV–infected patient, in complex with the receptor-binding domain of the SARS-CoV-2 spike. The antibody binds to an epitope conserved between SARS-CoV-2 and SARS-CoV that is distinct from the receptor-binding site. CR3022 likely binds more tightly to SARS-CoV because its epitope contains a glycan not present in SARS-CoV-2. Structural modeling showed that the epitope is only revealed when at least two of the three spike proteins are in a conformation competent to bind the receptor.

*Science*, this issue p. 630

## Teaser

Structural studies show that a SARS antibody binds a conserved epitope on the viral spike distinct from the receptor-binding site.

The ongoing outbreak of coronavirus disease 2019 (COVID-19) originated in China in December 2019 (*1*) and became a global pandemic by March 2020. COVID-19 is caused by a novel coronavirus, severe acute respiratory syndrome–coronavirus 2 (SARS-CoV-2) (*2*). Two other coronaviruses have caused worldwide outbreaks in the past two decades, namely SARS-CoV (2002–2003) and Middle East respiratory syndrome coronavirus (MERS-CoV) (2012–present). The surface spike (S) glycoprotein, which is critical for virus entry through engaging the host receptor and mediating virus-host membrane fusion, is the major antigen of coronaviruses. The S proteins of SARS-CoV-2 and SARS-CoV, which are phylogenetically closely related, have an amino acid sequence identity of ~77% (*3*). Such a high degree of sequence similarity raises the possibility that cross-reactive epitopes may exist.

**Fig. 1:**
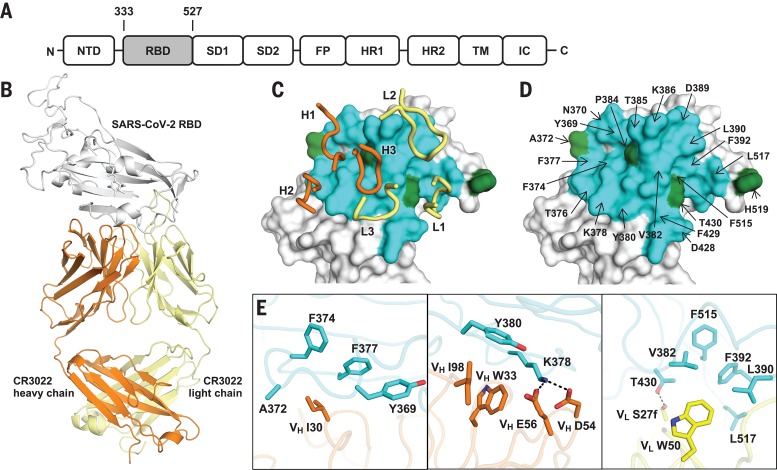
Crystal structure of CR3022 in complex with SARS-CoV-2 RBD. (**A**) Overall topology of the SARS-CoV-2 spike glycoprotein. NTD, N-terminal domain; RBD, receptor binding domain; SD1, subdomain 1; SD2, subdomain 2; FP, fusion peptide; HR1, heptad repeat 1; HR2, heptad repeat 2; TM, transmembrane region; IC, intracellular domain; N, N terminus; C, C terminus. (**B**) Structure of CR3022 Fab in complex with SARS-CoV-2 RBD. CR3022 heavy chain is orange, CR3022 light chain is yellow, and SARS-CoV-2 RBD is light gray. (**C** and **D**) Epitope residues on SARS-CoV-2 are shown. CDR loops are labeled. Epitope residues that are conserved between SARS-CoV-2 and SARS-CoV are shown in cyan, and those that are not conserved are shown in green. (D) Epitope residues that are important for binding to CR3022 are labeled. Epitope residues are defined here as residues in SARS-CoV-2 RBD with buried surface area > 0 Å^2^ after Fab CR3022 binding, as calculated with Proteins, Interfaces, Structures and Assemblies (PISA) (*34*). Single-letter abbreviations for the amino acid residues are as follows: A, Ala; D, Asp; E, Glu; F, Phe; H, His; I, Ile; K, Lys; L, Leu; M, Met; N, Asn; P, Pro; S, Ser; T, Thr; V, Val; W, Trp; and Y, Tyr. (**E**) Several key interactions between CR3022 and SARS-CoV-2 RBD are highlighted. CR3022 heavy chain is orange, CR3022 light chain is yellow, and SARS-CoV-2 RBD is cyan. Hydrogen bonds are represented by dashed lines.

CR3022, which was previously isolated from a convalescent SARS patient, is a neutralizing antibody that targets the receptor binding domain (RBD) of SARS-CoV (*4*). The immunoglobulin heavy chain variable, diversity, and joining (IGHV, IGHD, and IGHJ) regions are encoded by germline genes IGHV5-51, IGHD3-10, and IGHJ6, and the light chain variable and joining regions (IGKV and IGKJ) are encoded by IGKV4-1 and IGKJ2 (*4*). IgBlast analysis (*5*) indicates that the IGHV of CR3022 is 3.1% somatically mutated at the nucleotide sequence level, which results in eight amino acid changes from the germline sequence, whereas IGKV of CR3022 is 1.3% somatically mutated, resulting in three amino acid changes from the germline sequence (fig. S1). A recent study has shown that CR3022 can also bind to the RBD of SARS-CoV-2 (*6*). This finding provides an opportunity to uncover a cross-reactive epitope. We therefore determined the crystal structure of CR3022 with the SARS-CoV-2 RBD (Fig. 1A) at 3.1-Å resolution (table S1 and fig. S2, A and B) (*7*). CR3022 uses both heavy and light chains (Fig. 1B) as well as all six complementarity-determining region (CDR) loops (Fig. 1C) for interaction with the RBD. The buried surface area on the epitope is 917 Å^2^, and SARS-CoV-2 recognition by CR3022 is largely driven by hydrophobic interactions (Fig. 1E). Five out of 11 somatic mutations are found in the paratope region (defined as residues on the antibody buried by RBD) (fig. S2C), implying their likely importance in the affinity maturation process.

**Fig. 2:**
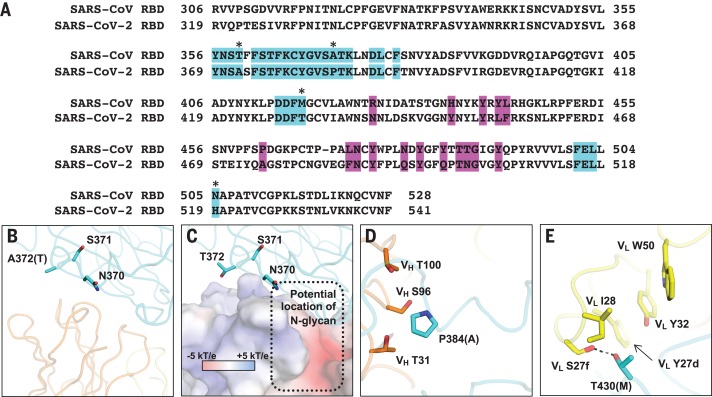
Conservation of epitope residues. (**A**) Sequence alignment of SARS-CoV-2 RBD and SARS-CoV RBD. CR3022 epitope residues are highlighted in cyan. ACE2-binding residues are highlighted in magenta. Nonconserved epitope residues are marked with asterisks. (**B** to **E**) Interactions between the nonconserved epitope residues and CR3022 are shown. Amino acid variants observed in SARS-CoV are in parentheses. SARS-CoV-2 RBD is cyan, CR3022 heavy chain is orange, and CR3022 light chain is yellow. Residues are numbered according to their positions on the SARS-CoV-2 S protein sequence. (B) Whereas SARS-CoV-2 has an Ala at residue 372, SARS-CoV has Thr, which introduces an N-glycosylation site at residue N370. (C) The potential location of N370 glycan in SARS-CoV RBD is indicated by the dotted box. CR3022 is shown as an electrostatic potential surface presentation with units of kT/e, where e is the charge of an electron, k is the Boltzmann constant, and T is temperature in kelvin. (D) P384 interacts with T31, S96, and T100 of CR3022 heavy chain. Ala at this position in SARS-CoV would allow the backbone to adopt a different conformation when binding to CR3022. (E) T430 forms a hydrogen bond (dashed line) with S27f of CR3022 light chain. Met at this position in SARS-CoV would instead likely insert its side chain into the hydrophobic pocket formed by Y27d, I28, Y32, and W50 of CR3022 light chain.

Out of 28 residues in the epitope (defined as residues buried by CR3022), 24 (86%) are conserved between SARS-CoV-2 and SARS-CoV (Figs. 1D and 2A). This high sequence conservation explains the cross-reactivity of CR3022. Nonetheless, despite having a high conservation of the epitope residues, CR3022 Fab binds to SARS-CoV RBD [dissociation constant (*K*_d_) = 1 nM] with a much higher affinity than it does to SARS-CoV-2 RBD (*K*_d_ = 115 nM) (Table 1 and fig. S3). The difference in binding affinity of CR3022 to SARS-CoV-2 and SARS-CoV RBDs is likely due to the nonconserved residues in the epitope (Fig. 2). The most drastic difference is an additional N-glycosylation site at N370 on SARS-CoV (N357 in SARS-CoV numbering). The N-glycan sequon (N-X-S/T, where X is any amino acid but proline) arises from an amino acid difference at residue 372, where SARS-CoV has a Thr compared with Ala in SARS-CoV-2 (Fig. 2B). Mass spectrometry analysis shows that a complex glycan is indeed present at this N-glycosylation site in SARS-CoV (*8*). An N-glycan at N370 would fit into a groove formed between heavy and light chains (Fig. 2C), which could increase contact and thus binding affinity to CR3022. This result also suggests that the difference in antigenicity between the RBDs of SARS-CoV-2 and SARS-CoV can be at least partially attributed to the N-glycosylation site at residue 370. We tested whether CR3022 was able to neutralize SARS-CoV-2 and SARS-CoV in an in vitro microneutralization assay (*7*). Although CR3022 could neutralize SARS-CoV, it did not neutralize SARS-CoV-2 at the highest concentration tested (400 μg/ml) (fig. S4). This in vitro neutralization result is consistent with lower affinity binding of CR3022 for SARS-CoV-2, although other explanations are also possible, as outlined below.

**Table 1:** Binding affinity of CR3022 to recombinant RBD and S protein. Binding affinity is expressed as the nanomolar dissociation constant (*K*_d_).

**Target**	**CR3022 IgG binding affinity (*K*_d_)**	**CR3022 Fab binding affinity (*K*_d_)**
SARS-CoV-2 RBD	<0.1	115 ± 3
SARS-CoV RBD	<0.1	1.0 ± 0.1

**Fig. 3:**
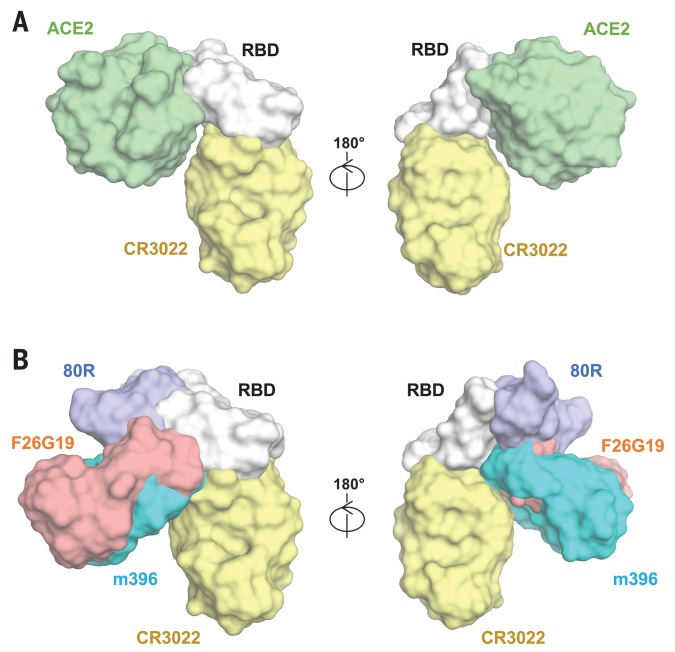
The relative binding location of CR3022 with respect to receptor ACE2 and other SARS-CoV RBD monoclonal antibodies. (**A**) Structures of CR3022–SARS-CoV-2 RBD complex and ACE2–SARS-CoV-2 RBD complex (*11*) are aligned on the basis of the SARS-CoV-2 RBD. ACE2 is green, RBD is light gray, and CR3022 is yellow. (**B**) Structural superposition of CR3022–SARS-CoV-2 RBD complex, F26G19–SARS-CoV RBD complex [Protein Data Bank (PDB) ID 3BGF] (*35*), 80R–SARS-CoV RBD complex (PDB ID 2GHW) (*36*), and m396–SARS-CoV RBD complex (PDB ID 2DD8) (*16*).

SARS-CoV-2 uses the same host receptor, angiotensin I–converting enzyme 2 (ACE2), as SARS-CoV (*3*, *9*–*11*). The epitope of CR3022 does not overlap with the ACE2-binding site (Fig. 3A). Structural alignment of the CR3022–SARS-CoV-2 RBD complex with the ACE2–SARS-CoV-2 RBD complex (*11*) further indicates that binding of CR3022 would not clash with ACE2 (*12*). This analysis implies that the neutralization mechanism of CR3022 for SARS-CoV does not depend on direct blocking of receptor binding, which is consistent with the observation that CR3022 does not compete with ACE2 for binding to the RBD (*6*). Unlike CR3022, most known SARS RBD-targeted antibodies compete with ACE2 for binding to RBD (*4*, *13*–*16*). The epitopes of these antibodies are very different from that of CR3022 (Fig. 3B). It has been shown that CR3022 can synergize with other RBD-targeted antibodies to neutralize SARS-CoV (*4*). Although CR3022 itself cannot neutralize SARS-CoV-2 in this in vitro assay, whether CR3022 can synergize with other SARS-CoV-2 RBD-targeted monoclonal antibodies for neutralization remains to be investigated.

**Fig. 4:**
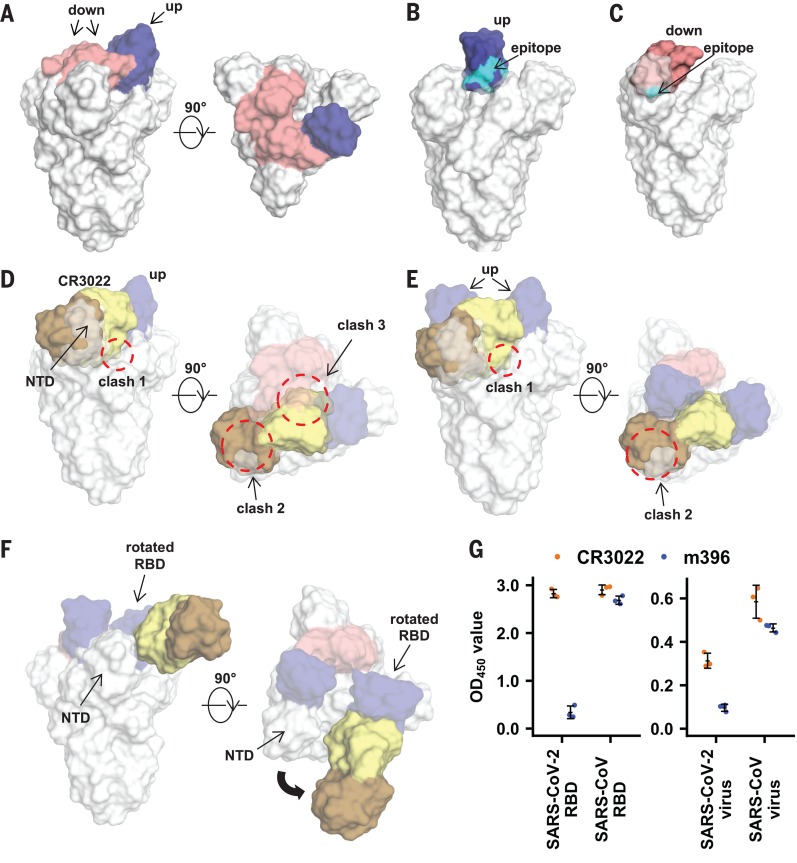
Model of the binding of CR3022 to the homotrimeric S protein. (**A**) RBD in the S proteins of SARS-CoV-2 and SARS-CoV can adopt either an up conformation (blue) or a down conformation (red). PDB ID 6VSB (cryo-EM structure of SARS-CoV-2 S protein) (*17*) is shown. (**B** and **C**) CR3022 epitope (cyan) on the RBD is exposed in (B) the up conformation but not in (C) the down conformation. (**D**) Binding of CR3022 to single-up conformation would clash (indicated by the red dashed circles) with the S protein. Clash 1: CR3022 variable region (yellow) clashes with the S2 domain. Clash 2: CR3022 constant region (brown) clashes with NTD. Clash 3: CR3022 variable region clashes with the neighboring RBD that is in the down conformation. (**E**) Clash 3 is resolved when the neighboring RBD is in the up conformation (i.e., S protein in double-up conformation). (**F**) All clashes are resolved if the targeted RBD is slightly rotated in the double-up conformation. The curved arrow indicates the change in CR3022 orientation due to the slight rotation of the ­RBD. Of note, given that the elbow angle between the constant and variable domains of CR3022 is the same as observed in our crystal structure, our model shows that a maximum rotation angle of ~45° for the RBD would avoid all clashes. However, the elbow region of an antibody is known to be highly flexible. Therefore, the rotation angle of the RBD could be much smaller when the spike trimer is bound to CR3022. (**G**) Binding of CR3022 immunoglobulin G (IgG) and m396 IgG to recombinant RBD proteins from SARS-CoV-2 and SARS-CoV (left panel) and to SARS-CoV-2 and SARS-CoV viruses (right panel). Black lines indicate mean ± standard deviation of three technical replicates. OD_450_, optical density at 450-nm wavelength.

The recent cryo–electron microscopy (cryo-EM) structures of the homotrimeric SARS-CoV-2 S protein (*17*, *18*) demonstrated that the RBD, as in other coronaviruses (*19*, *20*), can undergo a hinge-like movement to transition between “up” and “down” conformations (Fig. 4A). ACE2 host receptor can only interact with the RBD when it is in the up conformation—the down conformation is inaccessible to ACE2. The epitope of CR3022 is also only accessible when the RBD is in the up conformation (Fig. 4, B and C). However, even when one RBD in the SARS-CoV-2 S protein is in the up conformation, the binding of CR3022 to RBD can still be sterically hindered. Structural alignment of the CR3022–SARS-CoV-2 RBD complex with the SARS-CoV-2 S protein (*17*, *18*) indicates that the CR3022 variable region would clash with the RBD on the adjacent protomer if the latter adopted a down conformation. In addition, the CR3022 variable domain would clash with the S2 domain underneath the RBD, and the CR3022 constant region would clash with the N-terminal domain (Fig. 4D). Although, as compared with SARS-CoV-2, the up conformation of the RBD in SARS-CoV has a larger dihedral angle to the horizontal plane of the S protein (fig. S5), the clashes described above would also exist in the SARS-CoV S protein (fig. S6).

For CR3022 to bind to the S protein, the previously described clashes need to be resolved. The clash with the CR3022 variable domain can be partially relieved when the targeted RBD on one protomer of the trimer and the RBD on the adjacent protomer are both in the up conformation (Fig. 4E). SARS-CoV S protein with two RBDs in the up conformation has been observed in cryo-EM studies (*19*, *21*, *22*). Nevertheless, clashes with the N-terminal domain (NTD) and S2 domain would still exist in the “two-up” conformation. Further structural modeling shows that all clashes can be avoided with a slight rotation of the targeted RBD in the “double-up” conformation (Fig. 4F). This conformational change is likely to be physiologically relevant because CR3022 can neutralize SARS-CoV. In addition, our enzyme-linked immunosorbent assay (ELISA) experiment demonstrated that CR3022 is able to interact with the SARS-CoV-2 virus. Although the binding signals of CR3022 and m396, which is a SARS-CoV–specific antibody (*6*, *17*), to SARS-CoV were comparable in ELISA (*P* > 0.05, two-tailed *t* test) (Fig. 4G, left panel), CR3022 had a significantly higher binding signal to SARS-CoV-2 than did m396 (*P* = 0.003, two-tailed *t* test) (Fig. 4G, left panel), but not higher than its own binding signal to SARS-CoV, which is consistent with their relative binding to the RBD (Table 1 and fig. S3).

Our study provides insight into how SARS-CoV-2 can be targeted by the humoral immune response, and it reveals a conserved, but cryptic, epitope shared between SARS-CoV-2 and SARS-CoV. Recently, our group and others have identified a conserved epitope on influenza A virus hemagglutinin (HA) that is located in the trimeric interface and is only exposed through protein “breathing” (*23*–*25*), which is somewhat analogous to the epitope of CR3022. Antibodies to this influenza HA trimeric interface epitope do not exhibit in vitro neutralization activity but can confer in vivo protection. Similarly, antibodies to another conserved epitope that partially overlaps with the influenza HA trimeric interface are also non-neutralizing in vitro but protective in vivo (*26*). Examples of antibodies that do not have in vitro neutralization activity but confer in vivo protection have also been reported for influenza virus (*27*), herpesvirus (*28*), cytomegalovirus (*29*), alphavirus (*30*), and dengue virus (*31*). Therefore, although CR3022 does not neutralize SARS-CoV-2 in vitro, it is possible that this epitope can confer in vivo protection. Further study will require suitable animal models, which have yet to be established.

This coronavirus outbreak continues to pose an enormous global risk (*32*, *33*), and the availability of conserved epitopes may allow structure-based design not only of a SARS-CoV-2 vaccine but also of cross-protective antibody responses against future coronavirus epidemics and pandemics. Although a more universal coronavirus vaccine is not the most urgent goal at present, it is certainly worth future consideration, especially as cross-protective epitopes are identified, so that we can be better prepared for the next novel coronavirus outbreak.

## Supplementary Materials

science.sciencemag.org/content/368/6491/630/suppl/DC1 Materials and Methods Figs. S1 to S6 Tables S1 to S3 References (*37*–*46*) MDAR Reproducibility Checklist

## Appendix

View/request a protocol for this paper from *Bio-protocol*.
